# Analysis of the impact of urban summer high temperatures and outdoor activity duration on residents' emotional health: Taking hostility as an example

**DOI:** 10.3389/fpubh.2022.955077

**Published:** 2022-07-25

**Authors:** Huanchun Huang, Yang Li, Yimin Zhao, Wei Zhai

**Affiliations:** ^1^College of Landscape Architecture, Nanjing Forestry University, Nanjing, China; ^2^School of Architecture and Planning, The University of Texas at San Antonio, San Antonio, TX, United States

**Keywords:** outdoor activity duration, high temperature, emotional health, hostility, Beijing

## Abstract

The combined effect of global warming and the heat island effect keeps the temperature of cities rising in the summer, seriously threatening the physical and mental health of urban residents. Taking the area within the Sixth Ring Road of Beijing as an example, based on Landsat remote sensing images, meteorological stations, and questionnaires, this study established a relational model between temperature and hostility and then analyzed the changes in the emotional health risk (hostility) in the study area and the mechanism of how outdoor activity duration influences hostility. Results show that: (1) the area within the Sixth Ring Road of Beijing had a higher and higher temperature from 1991 to 2020. Low-temperature areas gradually shrank, and medium- and high-temperature areas extended outwards from the center. (2) The threat of high temperature to residents' hostility gradually intensified—the sphere of influence expanded, low-risk areas quickly turned into medium-high-risk areas, and the level of hostility risk increased. Level 1 risk areas of hostility had the most obvious reduction—a 74.33% reduction in area proportion; meanwhile, Level 3 risk areas had the most significant growth—a 50.41% increase in area proportion. (3) In the first 120 min of outdoor activities under high temperature, residents' hostility was negatively correlated with outdoor activity duration; after more than 120 min, hostility became positively correlated with duration. Therefore, figuring out how temperature changes influence human emotions is of great significance to improving the living environment and health level of residents. This study attempts to (1) explore the impact of temperature changes and outdoor activity duration on hostility, (2) evaluate residents' emotional health risk levels affected by high temperature, and (3) provide a theoretical basis for the early warning mechanism of emotional health risk and the planning of healthy cities.

## Introduction

At present, the frequent occurrence of extreme weather and global warming has brought significant impacts on the environment, ecosystems, society, and health, as well as pressing economic impacts related to labor, capital, goods, or services (1). In particular, increasingly severe urban summer high temperatures caused by urban heat islands has been widely treated as a major factor directly or indirectly threatening human health (2–4). Since the discovery of the urban heat island effect, scholars have conducted empirical research on the causes, influencing factors, and spatiotemporal distribution of urban high-temperature environments (5–7). Some studies have found that there is a correlation between urban morphological characteristics and functional area differences, and urban thermal environment, and at the same time, it has been proved that climatic zones also have an impact on urban surface temperature (8–12). Cities are the most important living areas of human beings, but they are also where disasters affect people's survival and daily lives the most. According to the latest data from the World Urbanization Prospects and World Population Prospects released by the United Nations Population Division, the world's urban population has exceeded 4.3 billion, accounting for about 55.3% of the total population, and this number may reach 68% by 2050 (13, 14). Urbanization continues to accelerate, and environmental problems caused by the urban heat island effect and global warming continue to deteriorate. Data shows that the global average temperature has risen by 1.1°C in the past century and may continue to rise in the future (15, 16). Faced with the dual pressures from accelerated urbanization and intensified heat island effect, how to improve the urban environment in order to protect the physical and mental health of residents has become a challenging issue worldwide. Hence, it is urgent to assess how the urban thermal environment threatens human health and establish an early warning mechanism.

The heat island effect exacerbates the harm brought by heatwaves and the risk of accidental death caused by heatstroke (17). The pooled relative risks of heatwaves on non-accidental mortality at lag 0, lag 0–2, and lag 0–10 days were 1.06 (95% CI: 1.03–1.09), 1.09 (1.05–1.13), and 1.10 (1.05–1.15), respectively. Compared with non-accidental mortality, higher effect estimates of heatwaves were observed among deaths from ischemic heart diseases, stroke, and respiratory diseases (18). It also increases the incidence of cardiovascular and cerebrovascular diseases and other heat-related diseases (e.g., heat cramps, and heat stroke) (19, 20). Previous studies indicate a significant correlation between elevated temperatures and the incidence of cardiovascular, cerebrovascular, and respiratory diseases (21, 22), and if the temperature exceeds a certain threshold, the regional excess mortality will increase (23–25). Studies on the impact of high temperature on human physical health have been bearing fruit. But according to the World Health Organization's definition, human health should also include spiritual and even social health. Unfortunately, up to now, few studies have explored the correlation between high temperature and people's emotional health. Current studies point out that high temperature and heat waves are significantly correlated with suicide, acute illness, severe depression, and admissions for mental illness (26, 27). The ambient temperature in summer has a significant impact on the length of hospital stay of patients with mental disorders, with two obvious thresholds—24.6°C and 33.1°C (28). A study using linear regression further proved that summer high temperatures severely affect the mortality associated with mental disorders (29). Studies have shown that a high ambient temperature also affects people's emotions, behavioral disorders, mental health, and other relevant health indexes (30). In addition, when the temperature exceeds certain thresholds, people's negative emotions intensify, even leading to a series of mental illnesses such as bipolar disorder (31, 32). According to Goal 3 (good health and wellbeing) of the Sustainable Development Goals Report 2021 issued by the United Nations, it is necessary to add the high-temperature effect as an important factor in safeguarding the health of people with mental or behavioral disorders.

The United Nations (UN) has included the promotion of subjective wellbeing (SWB) as one of the key indicators of its Sustainable Development Goals (SDGs) [(33, 92); UN-TOW]. Emotion is an important indicator of mental health and has an important impact on life wellbeing. Higher levels of positive emotions and lower levels of negative emotions can enhance high levels of life satisfaction (34, 35). Many studies have shown that emotions can be affected by environmental factors. Favorable soundscapes proved to be capable of reducing stress-related physiological markers; for example, nature sounds produce a higher reduction in skin conductance level than other sounds related to urban contexts (36). As a consequence, natural sounds were indicated as vital factors in easing stress recovery (37). The same visual and odor can also have an impact on people's emotions (38–40). The thermal environment has a more complex effect on emotional wellbeing (41). Compared to average daily temperatures in the 50–60 °F (10–16 °C) range, temperatures above 70°F (21°C) reduce positive emotions (e.g., joy, happiness), increase negative emotions (e.g., stress, anger), and increase fatigue (feeling tired, low energy) (42). At the same time, the length of exposure to high-temperature environments can also affect emotional health. Studies have found that prolonged heat exposure can seriously harm workers' physical and mental health, and work mood and further lead to a decline in productivity (43). Therefore, we wondered whether the duration of outdoor activities also had an emotion-related effect on the residents in the high-temperature environment.

Therefore, based on Landsat remote sensing images, electronic maps, meteorological data, and questionnaires collected by the research group, this study used ArcGIS, MATLAB, GraphPad, and other software for data processing and analysis, graded the summer high-temperature intensity, and analyzed the difference in its spatiotemporal evolution within the Sixth Ring Road of Beijing. Furthermore, this study decided on the emotion of hostility and took middle-aged and elderly people aged 40 and above as the study subjects to explore the relational model between residents' hostility level and two factors—urban summer high temperatures and outdoor activity duration. After that, this study evaluated the impact of high temperature on residents' emotional health. It provides a basis for carrying out emotional health risk early warning, and at the same time provides a reference for carrying out relevant urban planning adaptation measures.

## Materials and methods

### Overview of study area

Beijing, “Jing” for short, is the capital of the People's Republic of China, the political and cultural center of the country, at once both a world-famous ancient capital and a modern international metropolis. Beijing is located at 39°56′N, 116°20′E, in the northern part of the North China Plain, neighboring Tianjin city in the east, and Hebei province in the west, north, and south. The city is in the warm temperate zone, with a semi-humid and semi-arid monsoon climate, meaning hot and rainy in summer and cold and dry in winter. The terrain is high in the northwest and low in the southeast. The west, north, and northeast sides are surrounded by mountains, and the southeast is a plain gently tilting toward the Bohai Sea.

By the end of 2020, the permanent population of Beijing was 21.89 million, and the urbanization rate reached 87.6% (44). Under the influence of increased population density, a surge of anthropogenic heat, and changes in underlying surface attributes, the intensity of the heat island effect in Beijing increased with fluctuations that were significantly related to the city's urbanization progress (45).

**Figure 1:**
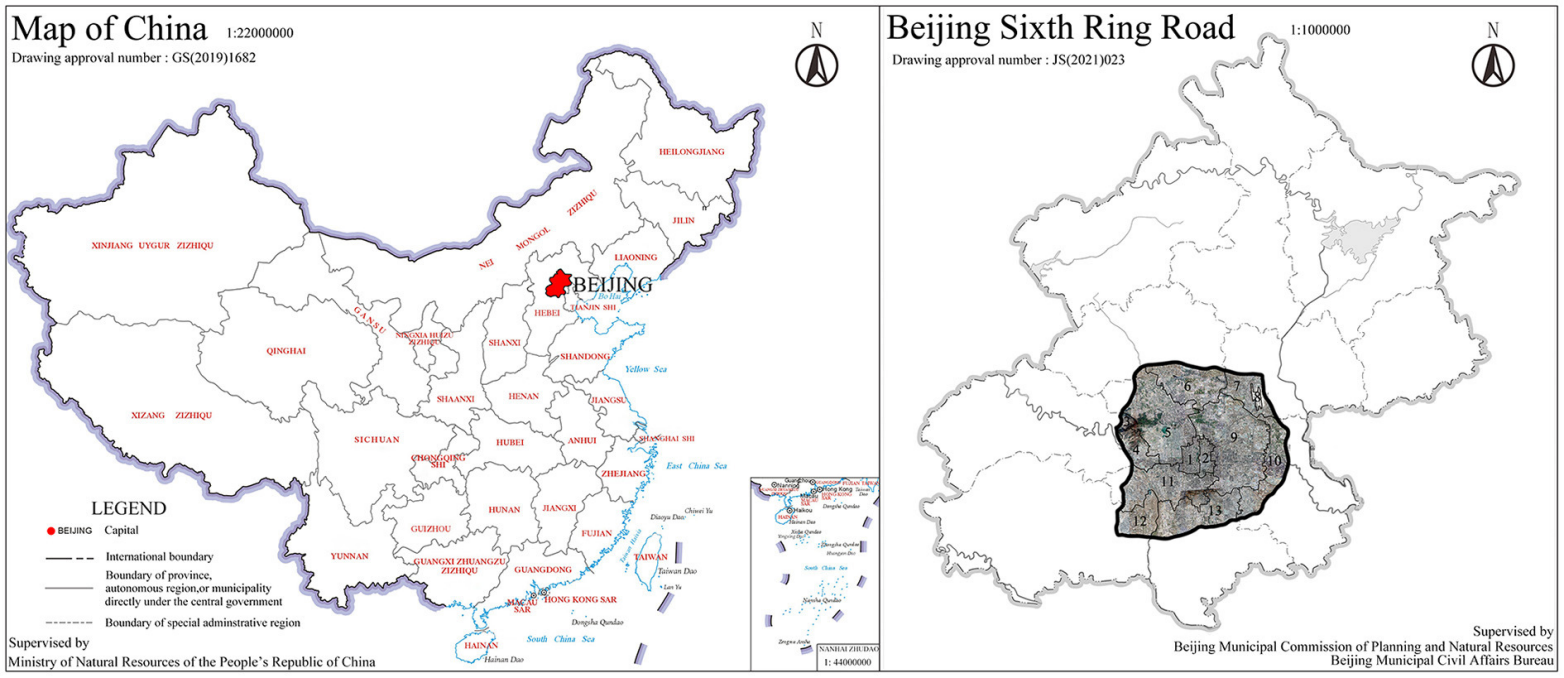
The location of study area (1: Xicheng District ; 2: Dongcheng District ; 3: Mentougou District ; 4: Shijinshan District ; 5: Haidian District ; 6: Changping District ; 7: Shunyi District ; 8: Beijing Capital International Airport ; 9: Chaoyang District ; 10: Tongzhou District ; 11: Fengtai District ; 12: Fangshan District ; 13: Daxing District).

This study took the most densely populated area—the area within the Sixth Ring Road (Dongcheng District, Xicheng District, Chaoyang District, Haidian District, Fengtai District, Shijingshan District, etc.) as the study area, covering a total area of 2,257.01 km^2^ (Figure 1).

### Data sources

#### Data collection

**Figure 2:**
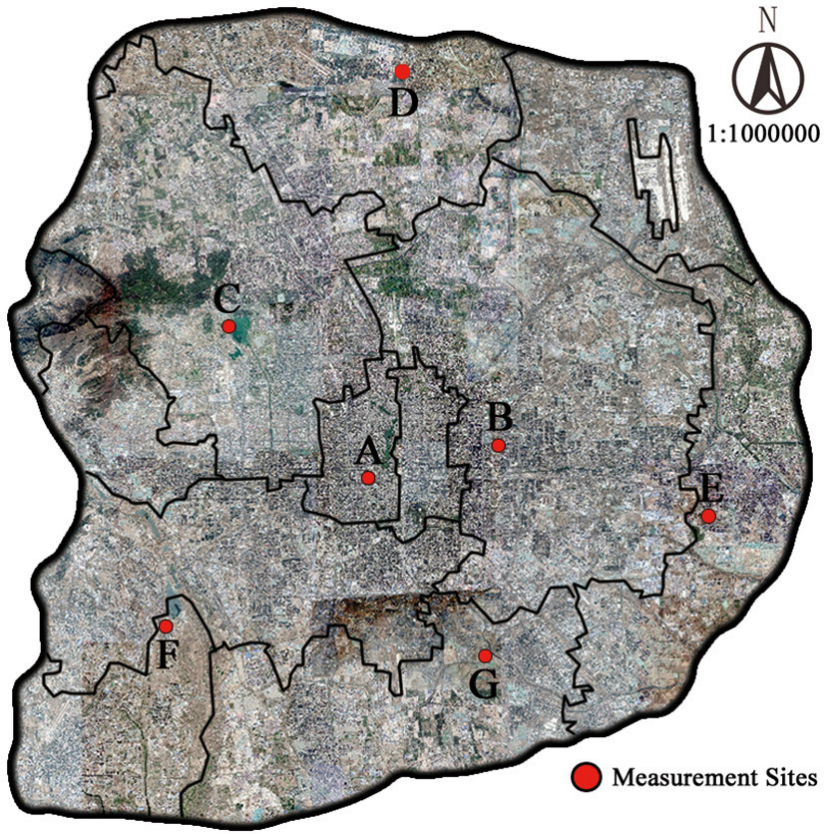
Measurement sites.

The data applied in this study include remote sensing images, meteorological data, and field survey questionnaires. Details are shown in Table 1. The remote sensing images with no cloud coverage, no precipitation, and wind speed below 2 m/s at the time of imaging were selected. The air temperature data was measured by the WS-30 small handheld weather station. The measurement accuracy of the device is ±0.3°C for temperature, ±3% for humidity, and ±0.3 m/s for wind speed. The device was placed at an observation point where buildings, vegetation, hardened ground, and artificial facilities around the measurement site had less interference with the heat island, and maintained a distance of 1.5 m from the ground. After the measurement data was stable, the weather station would automatically record data at 1-min intervals. There were 7 measurement sites (Figure 2 and Table 2), including park squares, commercial plazas, residential area squares, public squares, and other green open spaces for urban residents' daily life and activities, which were typically representative. July and August, the summer months with the highest temperature in Beijing, so the experiment dates were July 27-August 3, 2019 and August 1–3, 2020 to meet the high-temperature requirements for the study. At the same time, the surveys were conducted from 8:00 to 17:00 when the change in outdoor travel volume was relatively stable. When conducting research, we ensured that the sample was evenly distributed across time periods.

**Table 1:** Data sources.

**Type**	**Method**	**Time**	**Source**
Remote sensing image	Landsat 4/5/8	1991/1999/2011/2020	https://landsat.gsfc.nasa.gov/
Temperature	Meteorological station/ handheld weather station	2019/2020	Self-measurement
Hostility	Questionnaire	2019/2020	Self-measurement

**Table 2:** Survey location overview.

**Site**	**Type**	**Volume rate**
A	Public square	<0.2
B	Commercial center plaza	2.0–2.5
C	Park square	<0.1
D	Residential area square	1.5–2.0
E	Residential area square	1.5–2.0
F	Public square	<0.2
G	Commercial center plaza	2.5–3.0

#### Experimental procedure

According to the Positive and Negative Affect Schedule (PANAS) for Chinese people, improved by Huang et al. (46), this study used hostility as a proxy for measuring negative emotions. When the individual is threatened by the increase in the background temperature, he or she will have a hostile attitude and strong dissatisfaction, which is called hostile emotion, an extreme manifestation of negative emotion (47, 48). Face-to-face interviews were conducted through questionnaires asking respondents “How hostile do you feel now?” According to their current emotional state, respondents answered “1 almost none,” “2 relatively little,” “3 medium,” “4 relatively much” and “5 extremely much” to quantify their hostility. In addition to the hostility scale, gender, age, and time spent outdoors were also recorded.

**Figure 3:**
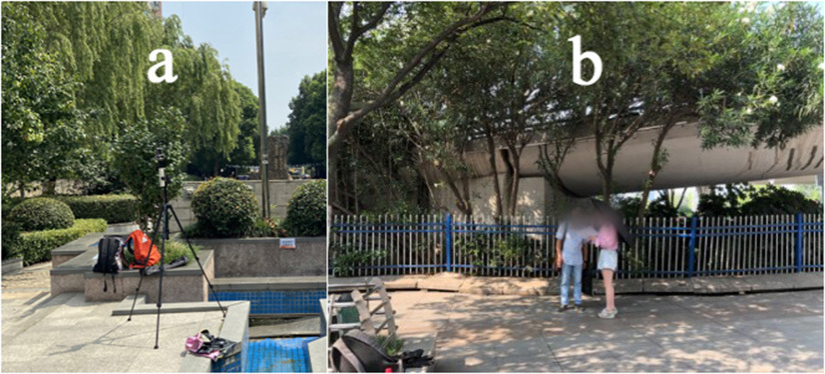
Temperature measurement and questionnaire [**(a)** handheld weather meter setup; **(b)** questionnaire distribution].

Questionnaires were handed out, and the temperature was measured at the same location at the same time (Figure 3), and the survey respondents were middle-aged and elderly people over 40 years old who were active outdoors. The questionnaires of this study are based on random sampling, so the influence of previous activities or unexpected events cannot be excluded from the research process. To minimize the bias of data analysis caused by these uncertainties, while ensuring the randomness of sampling in the meantime, we conducted a preliminary screening of the survey respondents, excluding those who just went out of indoor space or car, did strenuous exercise outdoors, and took a long rest in the shade.

## Result analysis

### Data processing

#### Sample data analysis

**Figure 4:**
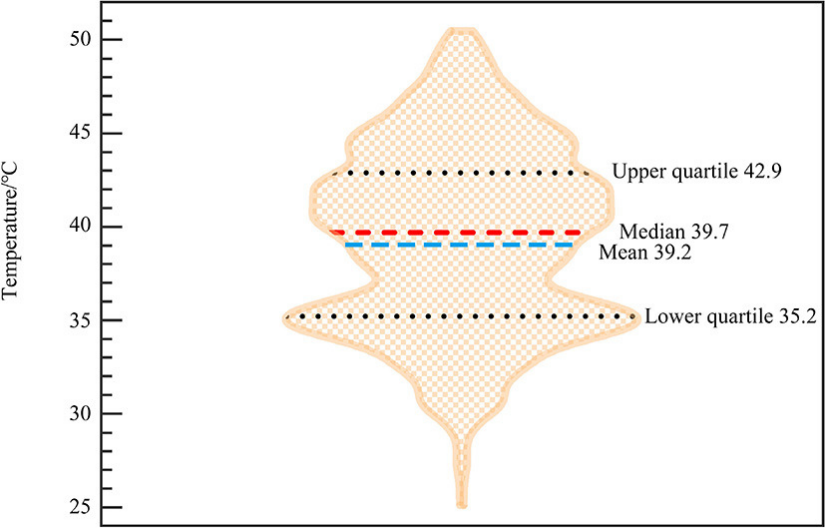
Sample temperature distribution.

The recorded air temperatures were typed into GraphPad for descriptive statistical analysis to draw a violin plot (Figure 4). Statistical analysis showed that the sample data covered 25.1–50.5°C with a relatively even distribution. The mean was 39.2°C, and the standard deviation was 4.89°C. The lower quartile was 35.2°C, the median was 39.7 °C, and the upper quartile was 42.9°C. The sample data was concentrated during 35.2–42.9°C, enough to cover the summer temperature range in the study area to analyze the effect of high temperature on hostility.

The questionnaire data were collected and summarized, and the questionnaire data pivot table (Table 3) was obtained. The results showed that the ratio of male to female respondents was ~1:1, and the difference in hostility between males and females was negligible. The mean age of the subjects was 64 years, and the subjects in the 40–49 age group had the highest mean hostility score at 1.45. Subjects' average outdoor activity time was 98 min, and subjects who were active for 1.5–2 h had the lowest hostility score of 1.22.

**Table 3:** Participant hostility and characteristics.

	**Count**	**Proportion (** ***n*** **%)**	**Mean**
Total	931	100	1.33
Gender			
Males	501	53.81%	1.33
Females	430	46.19%	1.33
Age			
40–49	104	11.17%	1.45
50–59	226	24.27%	1.43
60–69	329	35.34%	1.25
70–79	215	23.09%	1.31
≥80	57	6.12%	1.25
**Outdoor activity duration**			
<10 min	65	6.98%	1.30
10–20 min	66	7.09%	1.34
20–30 min	71	7.63%	1.30
30–40 min	50	5.37%	1.31
40–50 min	69	7.41%	1.29
50–60 min	113	12.14%	1.27
60–90 min	193	20.73%	1.26
90–120 min	79	8.49%	1.22
120–150 min	95	10.20%	1.24
150–180 min	51	5.48%	1.24
>180 min	79	8.49%	1.29

#### Temperature retrieval

Based on the Landsat satellite images, this study used the atmospheric correction method [also known as Radiative Transfer Equation (RTE)] for land surface temperature (LST) retrieval. The basic principles are: First, the influence of the atmosphere on the surface radiation is excluded from the total amount of thermal radiation received by the satellite sensor to obtain the surface brightness temperature, which was then converted into the corresponding LST (49, 50).

The hourly temperature field data in summer from meteorological stations were collected, and the hourly average temperature from 8:00 to 17:00 was used as the average daytime temperature. Regression analysis was carried out on LST, Normalized Vegetation Index (NDVI), and daytime average temperature, resulting in a linear regression equation with good significance (**Formula 1**).


(1) $$\begin{array}{l} T_{A}=0.448*T_{L}+1.454*y+15.729 \end{array}$$

where *T*_*A*_ is the average daytime temperature; *T*_*L*_ is LST; *y* is NDVI; and R^2^ = 0.576.

According to **Formula 1**, the raster data of the average daytime temperature of the imaging days in the 4 years was obtained *via* ArcGIS and then visualized. In this paper, the temperatures were divided into 6 levels—low-temperature area (≤27°C), lower-medium temperature area (27–29°C), medium temperature area (29–31°C), higher-medium temperature area (31–33°C), high-temperature area (33–35°C), and extreme-high temperature area (>35°C) (42).

#### Establishing a relational model between average daytime temperature and hostility

Based on the selected 931 questionnaires and temperature data from the handheld weather station, this study used SPSS for cross-tabulation, and the proportions of different degrees of hostility in each temperature range were obtained. The percentage of influence on hostility was weighted to get the integrated influence index in each temperature range. Finally, data were smoothed, and the maximum value of the influence index was normalized.

**Figure 5:**
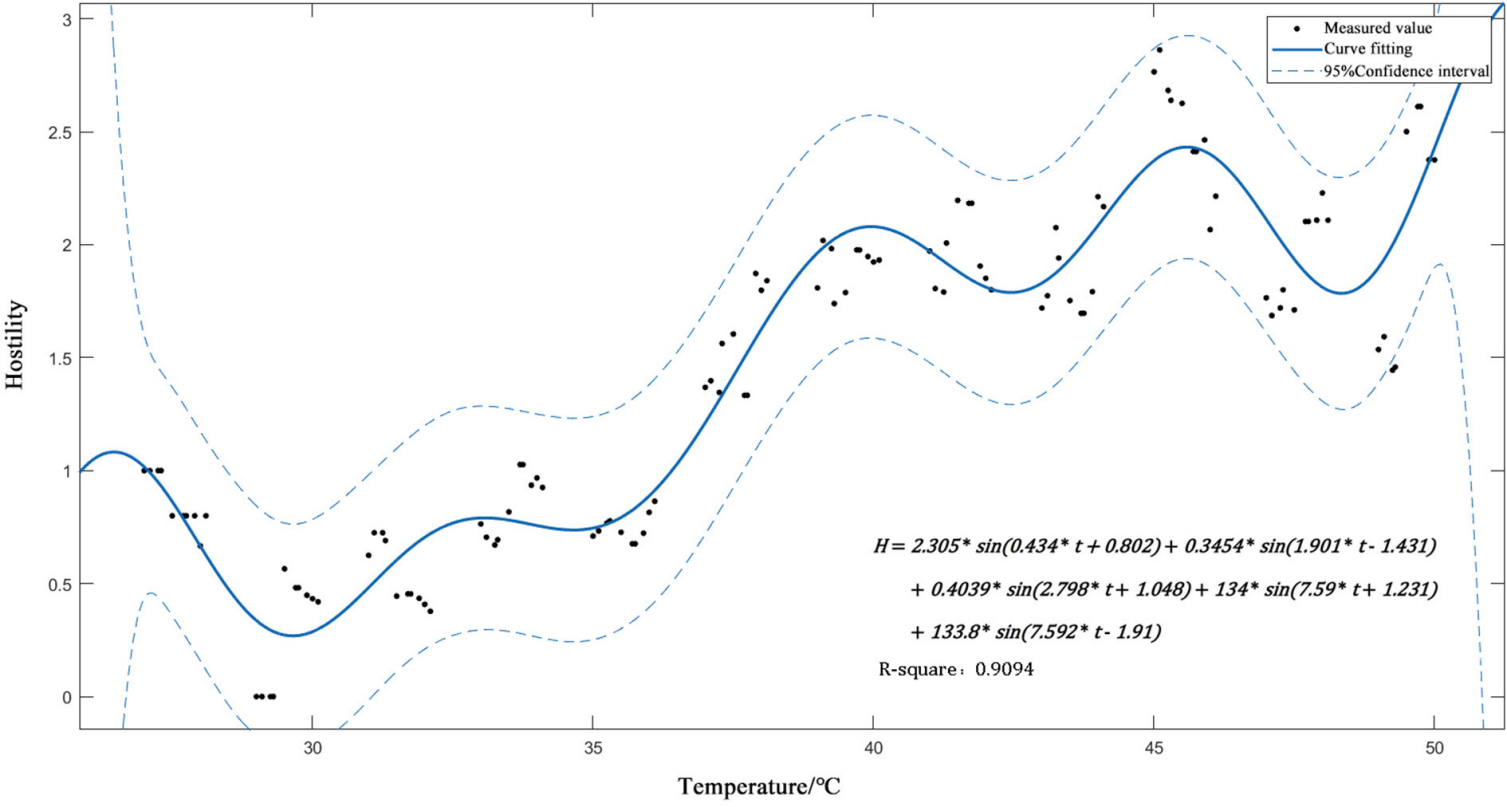
Relationship model between instantaneous temperature and hostility.

After that, this study used MATLAB to run multiple curvilinear regression analyses on the data, establishing a theoretical relational model between transient temperature and hostility and screening out the most fitted formula (**Formula 2**) (Figure 5).


(2) $$\begin{array}{l} H=2.305*sin(0.434*t+0.802)+0.3454\\ \;*sin(1.901*t-1.431)+0.4039*sin(2.798*t+1.048)\\ \;+134*sin(7.59*t+1.231)+133.8*sin(7.592*t-1.91) \end{array}$$

where *H* is the quantified hostility value; *t* is transient temperature. SSE = 5.904, R^2^= 0.9094.

**Figure 6:**
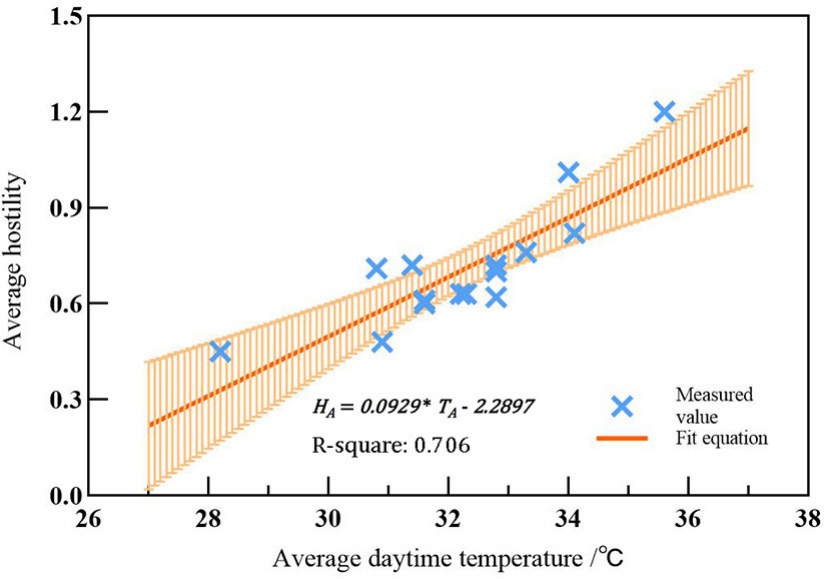
The relationship model between the average temperature during the day and the average hostility.

According to the relational model between transient temperature and hostility, the quantified hostility value from 8:00 to 17:00 every day was averaged as the average hostility value during the day, and the average temperature from 8:00 to 17:00 was taken as the average daytime temperature during the day, to establish a linear equation of the relationship between them (**Formula 3**, Figure 6).


(3) $$\begin{array}{l} H_{A}\;=\;0.0929\;*\;T_{A}\;-\;2.2897 \end{array}$$

where *H*_*A*_ is the average hostility value; *T*_*A*_ is the average daytime temperature. R^2^ = 0.706, Sy.x = 0.1072, with sound goodness of fit.

This study took the average daytime temperature of 27°C as the threshold, divided the degree of influence of urban high temperature on hostility into 6 levels, and used them as a standard for evaluating residents' emotional health risk. In detail, below 0.2 is Level 1 area, 0.2 to 0.4 Level 2 area, 0.4 to 0.6 Level 3 area, 0.6 to 0.8 Level 4 area, 0.8 to 1.0 Level 5 area, and above 1.0 Level 6 area (Table 4).

**Table 4:** Health risk assessment criteria for hostile emotions.

**Level**	**Average hostility value**	**Changes in hostility**
1	≤0.2	Comfortable
2	0.2–0.4	Emotionally stable
3	0.4–0.6	Slightly uncomfortable
4	0.6–0.8	Resentful
5	0.8–1.0	Prone to hostility
6	>1.0	Nervous, alert, easily provoked

### Analysis of the spatiotemporal evolution of summer high temperatures

The temperature within the Sixth Ring Road of Beijing has been significantly affected by urbanization in the past 20 years (51–53). The city was rapidly expanding outwards from the Forbidden City, and the concentrated population and buildings exacerbated the heat island effect. Analysis shows that from 1991 to 2020, the temperature changes were mainly concentrated in low-temperature areas (<27°C), lower-medium temperature areas (27–29°C), medium temperature areas (29–31°C) and higher-medium temperature areas (31–33°C). Meanwhile, high-temperature areas (33–35°C) and extreme-high temperature areas (>35°C) did not experience significant changes, and the area proportion changed by <0.5% (Table 5).

**Table 5:** Regional statistics of temperature changes within the Sixth Ring Road of Beijing.

**Temperature range**	**Year**							
	**1991**		**1999**		**2011**		**2020**	
	**Area/km** ^2^	**Proportion/%**	**Area/km** ^2^	**Proportion/%**	**Area/km** ^2^	**Proportion/%**	**Area/km** ^2^	**Proportion/%**
Low	1,179.42	51.99	225.55	9.94	16.89	0.74	13.23	0.58
Lower-medium	952.68	41.99	1,645.46	72.53	1,046.80	46.14	241.43	10.64
Medium	135.68	5.98	390.66	17.22	1,132.35	49.91	1,600.29	70.54
Higher-medium	0.81	0.04	6.56	0.29	71.24	3.14	408.18	17.99
High	0.01	0.00	0.35	0.02	1.30	0.06	5.25	0.23
Extreme-high	0.00	0.00	0.00	0.00	0.02	0.00	0.21	0.01


**Figure 7:**
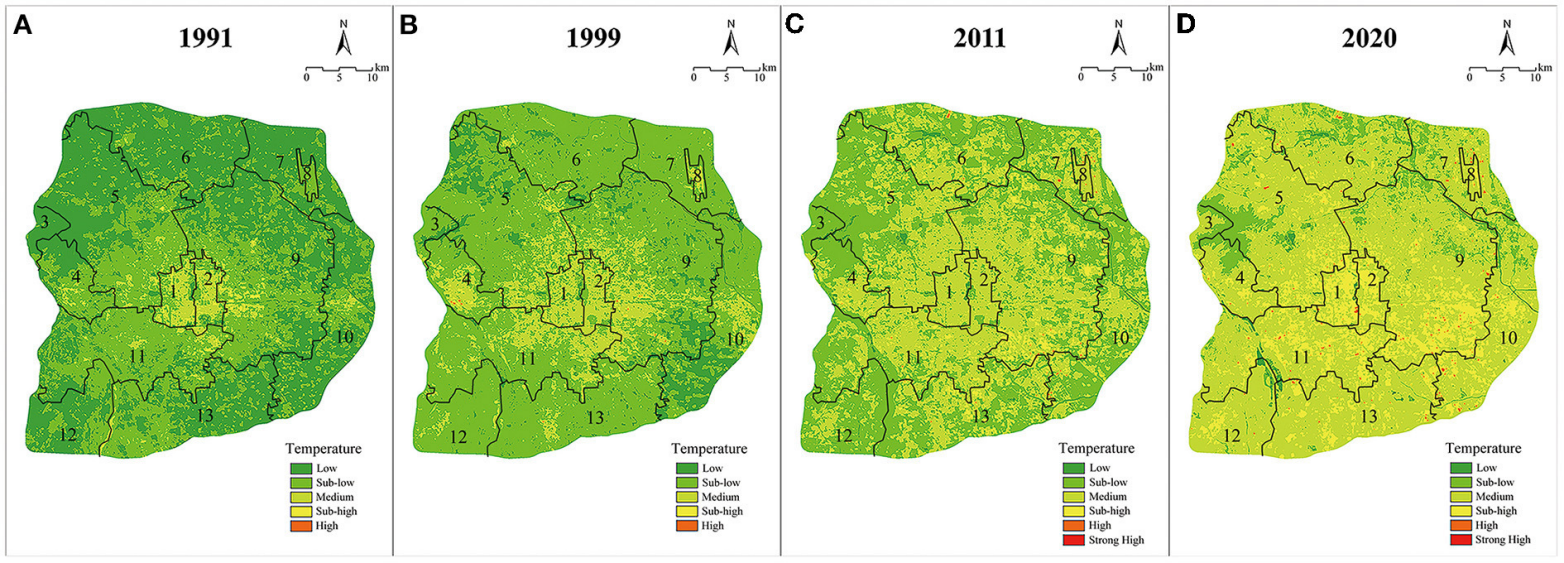
Spatial and temporal distribution of temperature of 1991 **(A)**, 1999 **(B)**, 2011 **(C)**, 2020 **(D)** (1: Xicheng District ; 2: Dongcheng District ; 3: Mentougou District ; 4: Shijinshan District ; 5: Haidian District ; 6: Changping District ; 7: Shunyi District ; 8: Beijing Capital International Airport ; 9: Chaoyang District ; 10: Tongzhou District ; 11: Fengtai District ; 12: Fangshan District ; 13: Daxing District).

In accordance with Figure 7, low-temperature areas experienced the most significant changes during the period between 1991 and 2020, with their proportion dropping by 51.41%, to <1% by 2020. In terms of spatial distribution, low-temperature areas were concentrated as large patches in the periphery in 1991, yet had dissipated by 1999 and then disappeared. Meanwhile, the total area of the lower-medium temperature areas increased by 72.72% from 1991 to 1999, expanding in a radial pattern from the center to the whole region. During urbanization, the outer suburbs were gradually heated up due to the heat island effect, transforming from low-temperature areas into lower-medium temperature areas, then rapidly dissipating. The medium temperature area, on the other hand, presented the most obvious trend of spreading. In 1999, the medium-temperature areas were concentrated in Dongcheng District and Xicheng Districts. In 2011, they began to spread radially from the central urban area to the periphery. From 2011 to 2020, they further penetrated into the outer suburbs. By 2020, they made up 70.54% of the total area of the study area. Next, higher-medium temperature areas went through a few changes from 1991 to 1999, with small broken patches scattered in the center. In 2011, it continued to expand to the periphery and gathered in small areas as broken patches. By 2020, continuous and small patches were concentrated in the urban built-up area. Last, high-temperature areas and extreme-high-temperature areas always maintained a very low proportion, less than 1%, showing a trend of slow growth.

### Assessment of the emotional health risk (hostility) of high temperature

**Figure 8:**
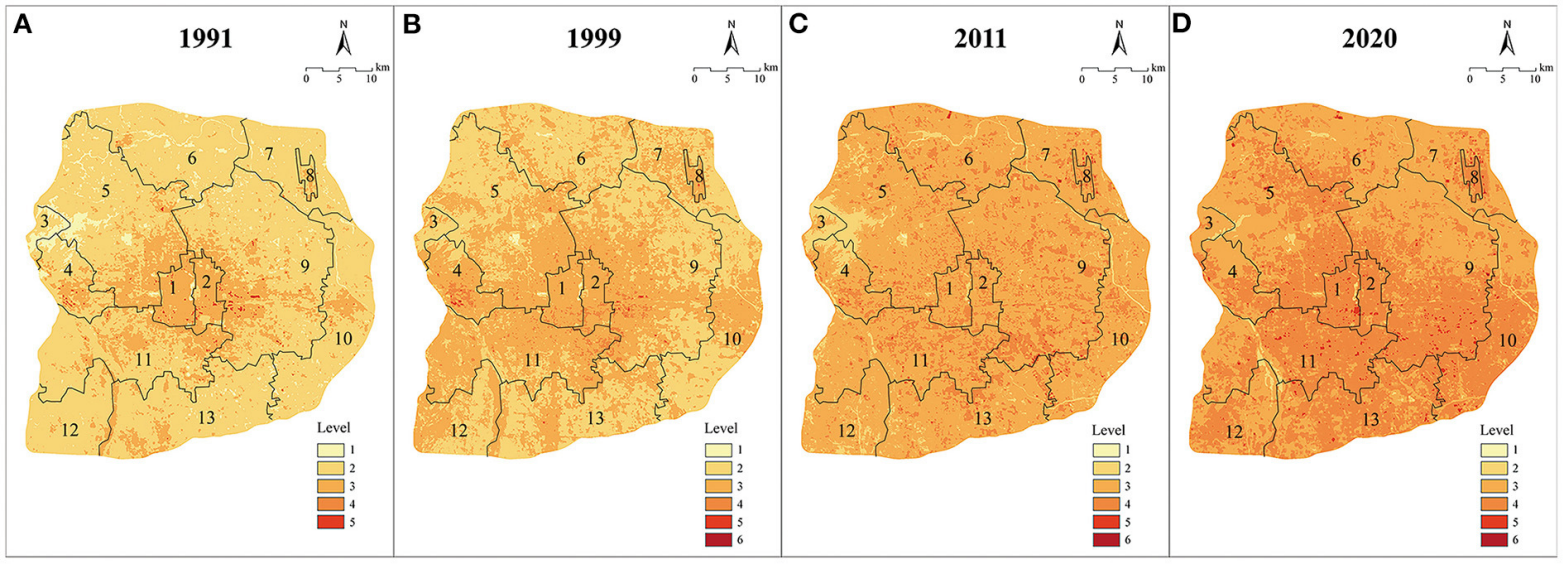
Hostility Health Heat Risk Level of year 1991 **(A)**, 1999 **(B)**, 2011 **(C)**, 2020 **(D)** (1: Xicheng District ; 2: Dongcheng District ; 3: Mentougou District ; 4: Shijinshan District ; 5: Haidian District ; 6: Changping District ; 7: Shunyi District ; 8: Beijing Capital International Airport ; 9: Chaoyang District ; 10: Tongzhou District ; 11: Fengtai District ; 12: Fangshan District ; 13: Daxing District).

This study evaluated the entire study area based on the standard for assessing the emotional health risk (hostility) of the high temperature mentioned above. Results showed (Table 6; Figure 8) that low-risk areas, consisting of Level 1 and Level 2 risk areas, dwindled rapidly in the study area. Eventually, only a few low-risk areas could be seen in the green space areas of Beihai Park, Zhonghai Park, Nanhai Park, Summer Palace, and Fragrant Hills Park. The remaining ones were less affected by urbanization due to their unique historical, cultural and ecological value, yielding little impact on the hostility of residents.

**Table 6:** Statistics on the emotional health risk level of hostility caused by high temperature within the Sixth Ring Road of Beijing.

Level	**Year**							
	**1991**		**1999**		**2011**		**2020**	
	**Area/km^2^**	**Proportion/%**	**Area/km^2^**	**Proportion/%**	**Area/km^2^**	**Proportion/%**	**Area/km^2^**	**Proportion/%**
1	94.84	4.18	5.80	0.26	0.68	0.03	0.65	0.03
2	1,714.59	75.58	1,063.52	46.88	152.74	6.73	28.26	1.25
3	452.45	19.94	1,146.56	50.54	1,753.13	77.28	1,063.56	46.88
4	6.62	0.29	51.57	2.27	356.71	15.72	1,150.16	50.70
5	0.09	0.00	1.13	0.05	5.24	0.23	25.44	1.12
6	0.00	0.00	0.02	0.00	0.09	0.00	0.53	0.02

After 30 years of development, medium-risk areas consisting of Level 3 and Level 4 areas occupied 97.58% of the study area. Level 3 risk areas expanded from the center to the surroundings, then to the whole region, rapidly increasing by 2.88 times from 1991 to 2011. Level 4 risk areas extended slowly during 1991-1999, scattered as a large number of dense patches in 2011, then as an interconnected matrix in 2020, with area proportion increasing from 0.29 to 50.70%. In terms of spatial distribution, the diffusion mode of Level 4 risk areas was not from the center to the surroundings. Instead, they started first in Shijingshan District, Chaoyang District, and Haidian District in the periphery of Dongcheng District and Xicheng District, later spreading to the whole region from 2011 to 2020.

High-risk areas (Level 5 and 6), affected mainly by the urban anthropogenic heat, changed slowly from 1991 to 2020, and gradually expanded from 2011 to 2020. Once the high-density urbanization development prevents the heat from dissipating into the air, high-risk areas easily expand and pose a greater threat to the emotional health of residents.

### Influence of residents' outdoor activity duration on hostility

**Figure 9:**
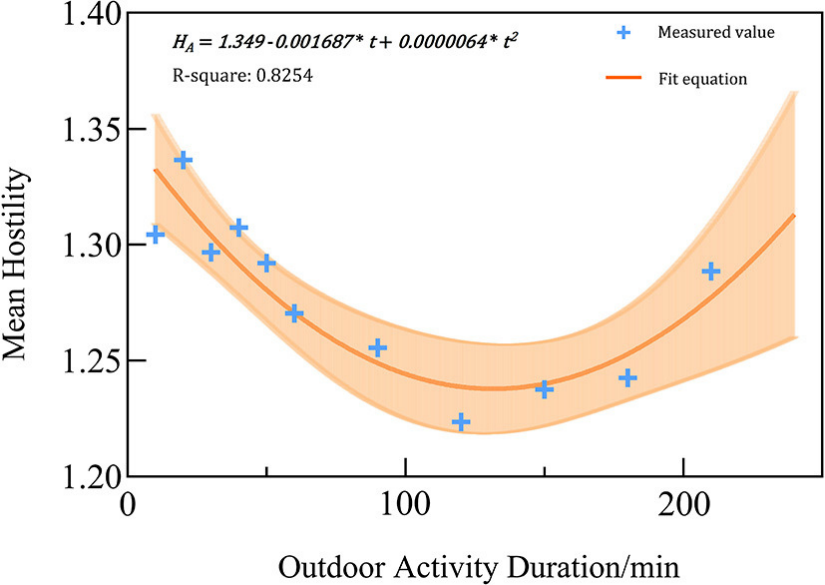
The trend of hostility with time of outdoor activities.

The average hostile emotion of subjects in each outdoor activity time period was counted, and a scatter plot was made with the outdoor activity time. The scatterplot showed a non-monotonic relationship between hostility and time spent outdoors (Figure 9). Therefore, the possibility of a linear correlation between the two was ruled out. In order to further explore how outdoor activity duration affects hostility under high temperatures, this study conducted linear and nonlinear regression analyses on the average hostility value and outdoor activity duration. Finally, a fitted nonlinear polynomial second-order least-squares fitting model was decided on **Formula 4**:


(4) $$\begin{array}{l} H_{A}\;=1.3490.001687*t+0.0000064*t^{2} \end{array}$$

where*H*_*A*_ is the quantified average hostility value, and *t* is the outdoor activity duration. R^2^ = 0.8254.

The regression equation showed a “U-shape” relationship between hostility and outdoor activity duration under high temperatures. As the duration lengthened, hostility first dropped significantly and then climbed up gradually—not exactly a positive correlation.

## Discussion

Overall, air temperature within the Sixth Ring Road of Beijing kept rising during the period from 1991 to 2020. Low-temperature areas gradually disappeared, and middle and high-temperature areas expanded outward from the center. This was inseparable from the rapid urbanization of Beijing. The heat island effect caused by rapid urbanization had a significant impact on the temperature increase in Beijing in summer (54–56). From 1985 to 2017, each 10% increase in the urbanization ratio with 10 km^2^ had led to additional annual warming of 0.14 ± 0.11, 0.17 ± 0.08, and 0.30 ± 0.17°C at nonrural stations for daily maximum (T-max), mean (T-mean), and minimum (T-min) air temperatures, respectively. Meanwhile, each 10% increase in the urbanization ratio with 10 km^2^ had led to additional changes of 11.6 ± 5.1, 12.7 ±- 5.8, and −11.1 ± 5.2 days at nonrural stations for hot days, hot nights, and chilly nights, respectively (51). Generally, urban areas tend to experience more severe heat stress under heat waves due to the urban heat island (UHI) effect (i.e., urban areas being warmer than rural areas) (57, 58). Increased solar radiation under heat waves was an important factor for the amplified UHIs. In addition, changes in wind direction also played an essential role (59). Human activities, including changes in the attributes of the underlying surface, reduction of green plants, and increase of heating elements like engines, made it so that the urban heat was unable to effectively be dissipated (60–63), increasing the urban temperature (64) and yielding a serious impact on the living environment of urban residents and threatening their physical and mental health (65–67).

Our study showed a positive correlation between hostility and average daytime temperature, when it exceeds 27°C, a finding that aligns with previous studies. Ambient temperatures above 70 °F, and especially above 90 °F, significantly reduce emotional well-being (42). And because of the rising summer temperatures in Beijing, the risk of heat waves to the emotional health of urban residents also continued to rise. The increasingly harsh urban thermal environment has caused serious harm to the physical and mental health of residents. Worse still, high temperatures brought residents greater stress and fatigue, a reduced sense of pleasure, and made them have psychological feelings like depression, anger, pain, and hostility, which in turn affected their mental health (68, 69). Contrary to the situation where high temperatures enhance negative emotions, negative emotions and mental fatigue are both relieved when temperatures are extremely low. Cold temperatures can reduce negative emotions by reducing feelings of aggression and arousal (70), which may also explain lower rates of violent crime in cold temperatures (71).

As shown in Figure 9, when outdoor activity lasted for <120 min, residents' hostility got alleviated gradually; when it lasted for over 120 min, residents' hostility climbed. When residents go from an air-conditioned indoor environment to a hot urban environment, it is difficult for them to quickly adjust to the temperature difference; therefore, they must focus on coping with the environmental threat (72, 73). The urban environment with high complexity, great spatial heterogeneity, and concentrated buildings can easily induce emotional reactions such as tension and hostility, and the external environment with low comfort can lead to excessive emotional stress response and intrinsic cognitive load (30, 74). As outdoor activity duration lengthened, the human body gradually adapts to the high-temperature environment, with hostility relieved (75). The “U-shape” relationship between residents' outdoor travel time and hostility is similar to the curve of physical health measures' response to temperature. Physical health measures respond negatively to both extreme cold and heat, with increases in negative outcomes observable at both ends of the temperature spectrum (76–78).

Although the high-temperature environment will harm people's emotional health and even physical and mental health (79), the human body also has a coping mechanism for high temperature, which can physiologically adapt to climate change to a certain extent (80). Studies have shown that people living in hotter climates at lower latitudes are more thermally adapted than people living in cooler climates at higher latitudes (81). In addition to the physiological heat adaptation, the human body also has an adaptation mechanism to high temperature (82), such that repeated heat exposure can improve the subject's sense of heat and sweat (83, 84). A more comprehensive understanding of the human body's adaptation mechanism to the thermal environment will help to formulate human adaptation strategies in the future.

In addition to the human body's thermal adaptability, the government can also take some active measures to adapt to the rising temperature and reduce the life hazards that may be caused by high-temperature heat waves. For example, identifying and warning of high-temperature health risk areas ahead of time, making preparations for the health system in advance, strengthening housing improvement and thermal adaptation urban planning and a series of policies (85, 86). Meanwhile, we should focus on socially vulnerable groups with poor heat adaptation (the elderly, those with low education levels, and patients with mental health problems, etc) (42, 87), and provide timely help and guidance, such as a direct policy recommendation stemming is for mental health providers to ensure patients get adequate sleep during a heat event (31).

The impact of high temperature on human emotional health is a complex process and a combined result of multiple factors (68). The questionnaire respondents of this study may experience emotional discomfort due to other uncontrollable objective factors such as humidity and wind speed. At the same time, the mental health level and group differences of these subjects would also interfere with the study, resulting in less valid or accurate data (88, 89). This study still has limitations by ignoring transient temperature, subjects' socioeconomic level and outdoor thermal experience, and other factors that may have affected the experimental results. Studies have shown that physiological indicators such as salivary cortisol, brain waves, and skin conductance levels can reflect changes in mental state (90, 91). In the future, more in-depth research on the relationship between physiological indicators and psychological and emotional health will help to better understand people's mental health and emotional health. At the same time, there are many factors that affect emotional health besides temperature, such as sound, vision, heat, smell, etc (36–38). The coupling effect of multiple factors on emotions can be explored in the future. This will aid in targeted urban planning as well as health policy.

## Conclusion

Based on various data from remote sensing images, meteorological stations, and questionnaires in Beijing, this study used ArcGIS, MATLAB and GraphPad for data processing to analyze the evolution trend of temperature in the study area from 1991 to 2020. In doing so, this study assessed the influence of the urban thermal environment on human emotional health (hostility) and the relationship between hostility and outdoor activity duration. Here are the conclusions:

(1) Affected by urbanization, air temperature within the Sixth Ring Road of Beijing kept rising over the past three decades. Low-temperature areas gradually disappeared, and medium and high-temperature areas showed a more and more obvious tendency to expand outwards from the center. The area of low and lower-medium-temperature areas dwindled by 82.76%, while that of medium and higher-medium-temperature areas increased by 82.51%.
(2) Influence of high temperature on hostility gradually intensified, and low-risk areas rapidly evolved into medium and high-risk areas, with the risk level of hostility increasing. Level 1 risk areas of emotional health (hostility) were cut down the most, with a reduction of 74.33% in the area proportion. Level 3 risk area, on the other hand, had the most obvious growth, with its area proportion climbing by 50.41%.
(3) Residents' hostility showed a “U-shape” relationship with outdoor activity duration in summer. In the first 120 min of outdoor activity, hostility is negatively correlated with duration; but when the activity lasts for more than 120 min, hostility becomes positively correlated with duration.

Against the backdrop of continuous global warming, the urban heat island effect gets intensified, and extreme weather conditions like extremely high temperatures happen more and more frequently year by year, yielding a serious and extensive impact on residents' emotional health. Hence, it is urgent to apply multi-level and multi-angle effective precautions (e.g., emotional health risk assessment and early warning) to better cope with climate changes in advance. In the future, while conducting in-depth research on how temperature affects human emotions and mental health, it is necessary to carry out urban planning, especially on residents' mental health, to help cities better adjust to climate changes and improve residents' overall health. In addition, we need to conduct interdisciplinary, integrated, and crossover research on climate, population, city, economy, and society to better cope with climate change and build healthy cities.

## Data availability statement

Publicly available datasets were analyzed in this study. This data can be found here: https://earthexplorer.usgs.gov/.

## Ethics statement

Ethics review and approval/written informed consent was not required as per local legislation and institutional requirements.

## Author contributions

HH: conceptualization, methodology, data curation, formal analysis, writing—original draft preparation, review and editing, investigation, and visualization. YL: methodology, data curation, formal analysis, writing—original draft preparation, review and editing, investigation, and visualization. YZ: validation. WZ: resources, data curation, and validation. All authors contributed critically to the manuscript and agreed to publication.

## Funding

The HH's work was supported the National Natural Science Foundation of China (Grant No. 319 31971717) and the Top-notch Academic Programs Project of Jiangsu (Grant No. PPZY2015A063).

## Conflict of interest

The authors declare that the research was conducted in the absence of any commercial or financial relationships that could be construed as a potential conflict of interest.

## Publisher's note

All claims expressed in this article are solely those of the authors and do not necessarily represent those of their affiliated organizations, or those of the publisher, the editors and the reviewers. Any product that may be evaluated in this article, or claim that may be made by its manufacturer, is not guaranteed or endorsed by the publisher.
